# Case Report: Hereditary thrombotic thrombocytopenic purpura mimicking immune thrombocytopenia: diagnostic pitfalls of whole-exome sequencing

**DOI:** 10.3389/fped.2026.1875502

**Published:** 2026-07-07

**Authors:** Nannan Xie, Yan He, Danlei Wu

**Affiliations:** Cangzhou People’s Hospital, Cangzhou, China

**Keywords:** ADAMTS13 gene, hereditary thrombotic thrombocytopenic purpura, immune thrombocytopenia, misdiagnosis, whole-exome sequencing

## Abstract

Herein, we describe a 22-month-old girl who presented with isolated severe thrombocytopenia (nadir platelet count: 6 × 10⁹/L) without overt microangiopathic hemolysis. The patient exhibited a partial clinical response to both corticosteroids and intravenous immunoglobulin (IVIG). Bone marrow examination showed increased megakaryocytes, with platelet-producing megakaryocytes accounting for 15% of the total megakaryocyte population. Initial whole-exome sequencing (WES) yielded negative findings for definitive pathogenic variants and clinically significant copy number variations (CNVs). Subsequent targeted genetic testing identified compound heterozygous ADAMTS13 variants: a paternally derived heterozygous exon 8 deletion validated by quantitative PCR (qPCR), and a maternally derived heterozygous frameshift mutation c.2764_2767dup (p.Ala923ValfsTer66) in exon 22 confirmed by Sanger sequencing. Functional analysis revealed severely diminished ADAMTS13 activity (0.23%) with undetectable inhibitory antibodies. The patient’s family history was remarkable for a first-trimester miscarriage and a previous neonatal death attributed to severe thrombocytopenia complicated by pulmonary hemorrhage. This case highlights two critical diagnostic pitfalls of pediatric hTTP: atypical presentation with isolated thrombocytopenia and misleading partial responsiveness to immunosuppressive therapy. In addition, routine WES has inherent limitations in detecting exon-level CNVs and certain frameshift variants. For children with unexplained recurrent thrombocytopenia and a suggestive familial bleeding history, ADAMTS13 functional assessment and targeted genetic sequencing should be performed promptly, even if initial WES results are unremarkable.

## 1 Introduction

Hereditary thrombotic thrombocytopenic purpura (hTTP) is a rare autosomal recessive disorder caused by biallelic mutations in the ADAMTS13 gene, with an estimated incidence of approximately one per million individuals (1). Deficiency of the ADAMTS13 protease leads to accumulation of ultra-large von Willebrand factor (vWF) multimers, which in turn triggers platelet microthrombus formation. The classic clinical pentad comprises microangiopathic hemolytic anemia, severe thrombocytopenia, neurological symptoms, renal impairment, and fever (2). However, hTTP in infants and young children frequently presents atypically. Isolated thrombocytopenia without overt hemolytic features is a common initial manifestation, and this atypical presentation often leads to misdiagnosis as ITP (3, 4). Additionally, some patients exhibit partial responses to corticosteroids and IVIG, further complicating the differential diagnosis. Although WES is widely employed for genetic diagnosis, its sensitivity for detecting CNVs and certain point mutations is limited, and pathogenic variants may be overlooked (5).

Herein, we describe a pediatric case of hTTP that was misdiagnosed as ITP for more than one year. The definitive diagnosis was ultimately established through additional genetic testing, targeted ADAMTS13 gene sequencing, and enzyme activity assays. This case highlights two major diagnostic pitfalls: false-negative WES results and atypical bone marrow morphology, both of which can substantially delay the diagnosis of hTTP in young children. We further analyze the contributing clinical factors, review recent updates on TTP phenotypic classification, and emphasize that targeted ADAMTS13 gene testing is indispensable when hTTP is clinically suspected, even when WES yields negative results.

## 2 Case description

### 2.1 Patient information

A 22-month-old female was admitted with a chief complaint of recurrent thrombocytopenia for more than one year, intermittent abdominal pain for one month, and cough with rhinorrhea for two days. At birth, she presented with severe thrombocytopenia (platelet count 19 × 10⁹/L) and diffuse petechiae and ecchymosis, without anemia. She received platelet transfusion, IVIG, and corticosteroid therapy with clinical improvement and was discharged on oral corticosteroids. Initial whole-exome sequencing (WES) was performed during the patient’s first hospitalization for neonatal thrombocytopenia and yielded negative pathogenic results. At four months of age, concurrent cytomegalovirus infection and an upper respiratory tract infection precipitated a decline in platelet count to 41 × 10⁹/L; she recovered following anti-infective treatment during hospitalization, after which corticosteroids were gradually tapered and discontinued. At nine months of age, a platelet count decrease occurred following DTaP vaccination; this resolved after IVIG administration. One month prior to the current admission, a further relapse was triggered by diarrhea and fever (platelet nadir 29 × 10⁹/L), during which hemoglobin decreased to a minimum of 101 g/L. She improved with IVIG, corticosteroids, platelet transfusion, and thrombopoietin (TPO), and was discharged on oral prednisone 10 mg/day (approximately 1 mg/kg/day) (Table 1).

**Table 1:** Clinical timeline.

Time point	Clinical event	Platelet (× 10⁹/L)	Hgb (g/L)	Treatment	Response
Birth (0 months)	Initial onset	19	144	Platelet transfusion, IVIG, glucocorticoids; oral methylprednisolone after discharge	Effective
4 months	CMV+URTI	41	106	IVIG, anti-infective therapy; steroids tapered	Effective
9 months	Relapse after vaccination	6	104	Platelet transfusion, IVIG	Effective
∼21 months (1 month pre-admission)	Relapse after diarrhea/fever	7 (nadir 29)	106	IVIG, corticosteroids, platelet transfusion, TPO; oral prednisone after discharge	Effective
Week −2 (outpatient)	Outpatient review	29	101	Prednisone 10 mg/day	—
Day 0 (admission)	Hospital admission	65	119	Prednisone 10 mg/day	—
Day 1	Bone marrow aspiration	95	119	Prednisone 10 mg/day	Good response
Week 1 post-admission	Outpatient follow-up	172	117	Maintenance prednisone	Normalized
Week 2 post-admission	Outpatient follow-up	142	125	Prednisone tapered to 5 mg/day	Stable
Week 3 post-admission	Outpatient follow-up	171	127	Prednisone 5 mg/day	Stable
Post-discharge (ongoing)	hTTP diagnosis confirmed; steroid taper	30–105 (fluctuating)	Stable	Oral prednisone gradually tapered/discontinued; FFP on demand (platelet <20 × 10⁹/L or surgical/trauma risk)	Partial, sustained; no full normalization; consistent with compensated hTTP without enzyme replacement

**Family history:** The mother had three pregnancies. The first ended in a first-trimester miscarriage. The second child died in the neonatal period due to severe thrombocytopenia and pulmonary hemorrhage. The proband is the third child. Both parents are healthy, non-consanguineous, and have no personal history of bleeding disorders.

### 2.2 Laboratory examinations

Lactate dehydrogenase (LDH) and reticulocyte counts were mildly elevated. Intermittent mild anemia was present; however, hyperbilirubinemia and schistocytes were absent, and there were no typical features of microangiopathic hemolysis. Autoantibody profiles and immunoglobulin levels were within normal ranges. Liver function, renal function, and coagulation studies were unremarkable. Serological testing for cytomegalovirus and Epstein–Barr virus was negative. In light of the persistent family history and steroid-dependent thrombocytopenia, we performed targeted gene reanalysis and simultaneously conducted ADAMTS13 activity assays and anti-ADAMTS13 inhibitory antibody testing (see Section 3.3).

### 2.3 Bone marrow examination

**Figure 1:**
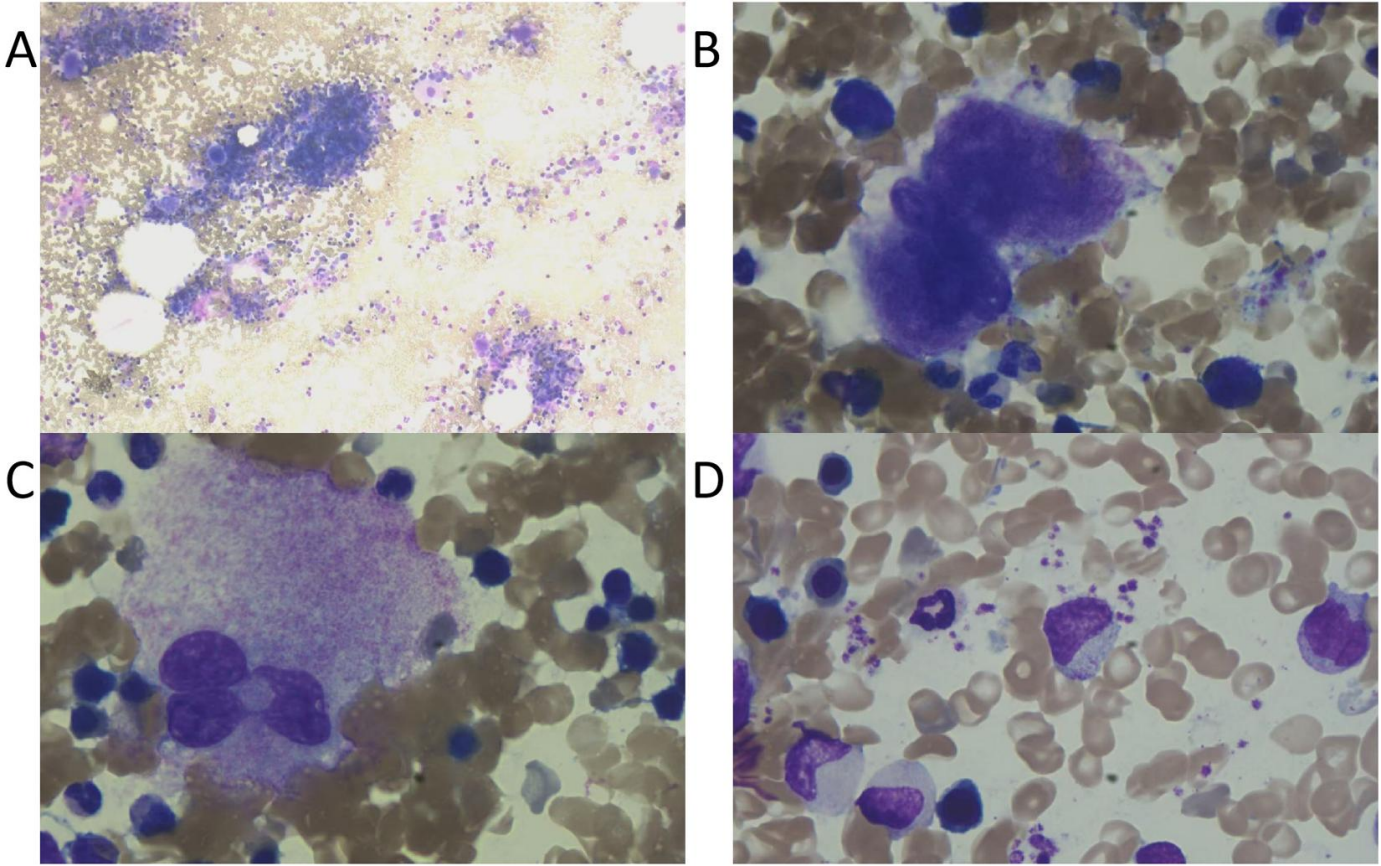
Bone marrow aspirate morphology. **(A)** Low-power view (×  100) showing megakaryocyte proliferation; **(B)** High-power view (×  1,000) showing platelet-producing megakaryocytes; **(C)** High-power view (×  1,000) showing granular megakaryocytes; **(D)** Platelet clusters visible on the smear.

On Day 2 of admission (during ongoing corticosteroid therapy), bone marrow aspiration was performed. The marrow exhibited active cellularity with a granulocyte-to-erythroid (G/E) ratio of 1.5:1. The proportions and morphology of both granulocytic and erythroid lineages were unremarkable. A total of 114 megakaryocytes were identified: 30% immature megakaryocytes, 55% granular megakaryocytes, 15% platelet-producing megakaryocytes, and 0% bare-nucleus megakaryocytes. Platelet clusters were also observed. (Figure 1).

### 2.4 Treatment course

Prior to admission, the patient was maintained on oral prednisone acetate 10 mg/day without platelet transfusion. Platelet counts progressively increased following admission, and the dose was reduced to 5 mg/day one week after discharge. The child maintained satisfactory general condition without hemorrhagic events or adverse reactions. The apparent efficacy of corticosteroid and IVIG therapies, in combination with the initially negative WES result, constituted the principal factors contributing to diagnostic delay. The mechanistic basis for this apparent treatment response and its distinction from true immunosuppressive efficacy are discussed in Section 4.1.

## 3 Genetic analysis

### 3.1 Initial whole-exome sequencing (WES)

WES (blood system panel, 500 ×   coverage) combined with mitochondrial genome sequencing was performed at an accredited reference laboratory approximately one year before the current admission. Samples were collected during the patient’s first hospitalization for neonatal thrombocytopenia. The report identified no pathogenic or likely pathogenic variants accounting for the clinical phenotype. No clinically significant CNVs or pathogenic mitochondrial gene variants were detected.

### 3.2 Targeted supplementary sequencing and variant confirmation

Genomic DNA was extracted from peripheral blood leukocytes of the proband and her parents. Whole-exome sequencing (WES) was performed on the Illumina NovaSeq 6000 platform with an average sequencing depth of 500 ×   to screen for potential pathogenic variants associated with thrombocytopenia. For validation of the suspected exon 8 copy number variation (CNV) of the ADAMTS13 gene, quantitative real-time polymerase chain reaction (qPCR) was conducted using the ABI 7500 Real-Time PCR System, and the copy number was calculated via the *ΔΔ*Ct method. Sanger sequencing was applied to verify the point variant and familial segregation: target gene fragments were amplified by PCR, followed by sequencing on the ABI 3730xl DNA Analyzer, and sequence alignment and variant analysis were performed using Chromas software.

Initial WES failed to detect the exon 8 CNV of ADAMTS13 and yielded false-negative results, primarily due to the insufficient sensitivity of routine WES pipelines for exon-level copy number variations and relatively low local target region coverage. Given that the patient developed thrombocytopenia immediately after birth and has a persistent family history of bleeding disorders, these findings strongly suggest a potential genetic etiology. We adopted a multi-layered secondary genetic re-analysis strategy: optimized CNV pipeline re-analysis of raw WES data, supplementary targeted ADAMTS13 panel sequencing, and orthogonal validation via qPCR and trio-Sanger familial segregation testing.

**Figure 2:**
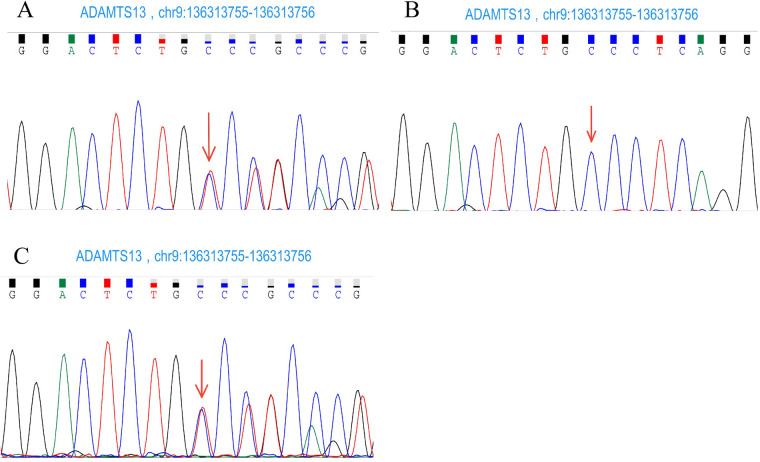
Sanger sequencing chromatograms (exon 22, c.2764_2767dup). **(A)** Proband (26C155750): heterozygous duplication variant. **(B)** Father (26C155751): no variant at this locus (wild type). **(C)** Mother (26C155752): heterozygous duplication variant confirming maternal inheritance. The exon 8 deletion was confirmed by qPCR; representative copy-number data are provided in the Supplementary Material.

Two compound heterozygous ADAMTS13 variants were identified and confirmed: (1) Exon 8 heterozygous deletion (paternal allele): A heterozygous deletion spanning the genomic region chr9:136294025–136295981 (NM_139027.6, exon 8) was detected by WES. This CNV was subsequently validated in the proband and confirmed in the father by qPCR, targeting four contiguous amplicons across the deleted region (chr9:136294800–136295350), all of which demonstrated a copy number ratio consistent with heterozygous deletion. We acknowledge that multiplex ligation-dependent probe amplification (MLPA) is the gold standard for CNV validation; however, convergent findings from WES copy-number analysis and multi-amplicon qPCR, combined with clear parental segregation data, provide robust orthogonal evidence for pathogenicity. According to ACMG/AMP criteria, this deletion is classified as Pathogenic (PVS1: predicted loss of function by exon deletion; PM2_Supporting: absent from population frequency databases; PM3: detected in trans with a second pathogenic variant in a recessive disorder). To our knowledge, this specific exon 8 deletion breakpoint has not been previously reported in the literature or in ClinVar, thereby expanding the known mutational spectrum of ADAMTS13. (2) Exon 22 heterozygous frameshift variant (maternal allele): The variant c.2764_2767dup (p.Ala923ValfsTer66) in exon 22 (NM_139027.6) was identified by WES and confirmed by trio Sanger sequencing; the duplication was present in both the proband and the mother, and absent in the father (Figure 2). This frameshift introduces a premature stop codon predicted to trigger nonsense-mediated mRNA decay (NMD), resulting in complete loss of functional ADAMTS13 from the maternal allele. According to ACMG/AMP criteria, this variant is classified as Likely Pathogenic (PVS1: frameshift causing predicted NMD; PM2_Supporting: absent from population databases). This variant has not been previously reported in ClinVar or the published literature, representing a novel ADAMTS13 mutation.

Together, these compound heterozygous variants provide definitive molecular confirmation of hereditary TTP (OMIM: 274150) in this patient.

### 3.3 ADAMTS13 activity and inhibitory antibody testing

ADAMTS13 activity was severely reduced at 0.23% (normal range: 40%–130%). ADAMTS13 inhibitory antibodies were negative (6.77 U/mL; reference: <12 U/mL). These results effectively exclude acquired immune TTP (iTTP) and, in conjunction with the confirmed biallelic ADAMTS13 variants, establish the diagnosis of hereditary TTP (Table 2).

**Table 2:** ADAMTS13 functional detection.

Gene	Variant	Zygosity	ACMG classification	Validation	Inheritance
ADAMTS13	chr9:136294025–136295981; exon 8 deletion	Heterozygous	Pathogenic (PVS1 + PM2 + PM3)	WES+qPCR	Paternal
ADAMTS13	c.2764_2767dup; p.Ala923ValfsTer66; exon 22	Heterozygous	Likely Pathogenic (PVS1 + PM2)	WES+Sanger	Maternal

### 3.4 Family perspectives and genetic counseling

The parents reported that their first child died three days after birth from pulmonary hemorrhage of unexplained etiology at the time. Following the proband’s first episode of recurrent thrombocytopenia, the family underwent multiple hospitalizations spanning more than one year; each episode showed partial response to IVIG and corticosteroids, yet symptoms recurred. Molecular confirmation of the underlying diagnosis ultimately provided an explanation for the family’s adverse reproductive outcomes and enabled informed reproductive planning. Written informed consent for publication was obtained from the legal guardian.

Genetic counseling confirmed that both parents are asymptomatic heterozygous carriers, with a 25% recurrence risk per pregnancy. Preventive strategies include prenatal diagnosis or preimplantation genetic testing (PGT-M). For subsequent at-risk pregnancies, prophylactic transfusion of fresh frozen plasma (FFP) is recommended for the newborn immediately after birth.

## 4 Discussion

### 4.1 Diagnostic pitfalls, overlap syndrome consideration, and mechanistic analysis

In this case, the patient was misdiagnosed with ITP for more than one year. Four key factors contributed to this diagnostic error.

First, Children with hereditary TTP frequently present with recurrent isolated thrombocytopenia without classic microangiopathic hemolysis, which is commonly misdiagnosed as immune thrombocytopenic purpura, leading to long-term diagnostic delay (3, 4, 6, 7).

Second, apparent response to corticosteroids and IVIG. We thoroughly evaluated the possibility of an overlap syndrome, including acquired iTTP, concomitant ITP, or the recently described ‘unidentified TTP’ (uTTP)—a putative third form of TTP characterized by severe ADAMTS13 deficiency in the absence of detectable anti-ADAMTS13 antibodies and without confirmed biallelic ADAMTS13 mutations (8–10). However, several independent lines of evidence collectively exclude these alternatives: (a) ADAMTS13 activity was severely and persistently reduced at 0.23%, far below the 10% diagnostic threshold; (b) ADAMTS13 inhibitory antibodies were definitively negative; (c) compound heterozygous biallelic pathogenic ADAMTS13 variants were confirmed by multiple orthogonal methods with clear parental segregation; and (d) the family history of recurrent neonatal hemorrhagic death and early pregnancy loss is a hallmark of heritable, rather than acquired, disease. The apparent corticosteroid and IVIG responsiveness most likely reflects non-immunologic mechanisms. Glucocorticoids stabilize vascular endothelial cell integrity, suppress pro-inflammatory cytokines including TNF-*α* and IL-1*β*, and exert modulatory effects on megakaryopoiesis (11, 12), Although these effects can temporarily elevate peripheral platelet counts, they fail to correct the underlying congenital ADAMTS13 enzyme deficiency, resulting in incomplete and non-sustained therapeutic responses. After gradual steroid tapering following discharge, the patient’s platelet counts remained stably fluctuating at 30–105 × 10⁹/L without complete normalization. This partial and unstable hematological recovery is consistent with the state of partially compensated hTTP, rather than typical ITP remission, in which full platelet count restoration is expected. Special caution should be exercised regarding platelet transfusion in TTP management, as this intervention is conventionally considered risky due to its potential to exacerbate platelet-rich microthrombosis and aggravate microangiopathy. Nevertheless, no clinical deterioration, worsening hemolysis, or new ischemic complications were observed after platelet transfusion in this patient. This benign post-transfusion outcome can be attributed to the unique hypoprothrombotic physiological microenvironment in infants. Neonatal platelets exhibit inherent hyporeactivity and impaired aggregation function, which prevents excessive activation driven by elevated ultra-large von Willebrand factor multimers. Meanwhile, immature endothelial stress and inflammatory responses in early infancy further limit the progression of microvascular injury. Collectively, these developmental hemostatic characteristics narrow the functional gap between residual ADAMTS13 activity and the threshold for overt thrombotic microangiopathy, thereby mitigating the typical fulminant TMA phenotype in pediatric hTTP.

Bone marrow examination revealed increased megakaryocytes with 15% platelet-producing megakaryocytes, and absence of schistocytes or active erythroid proliferation were observed (see Section 2.3). Classic ITP is characterized by compensatory bone marrow responses secondary to immune-mediated platelet destruction, typically presenting with normal or elevated megakaryocyte counts dominated by immature and granular megakaryocytes, markedly reduced platelet-producing megakaryocytes, and scarce platelets surrounding megakaryocytes. In contrast, the patient exhibited only a mild reduction in platelet-producing megakaryocytes with clustered platelets adjacent to megakaryocytes. These morphological features were inconsistent with the typical bone marrow manifestations of ITP and also did not conform to classic TTP-associated myelographic changes. The atypical morphological findings were presumed to be attributable to glucocorticoid-mediated modulation of megakaryocyte maturation and platelet release (11). However, this interpretation remains speculative due to the absence of baseline bone marrow data obtained prior to steroid treatment. Collectively, these unusual marrow features, combined with the presentation of isolated thrombocytopenia and transient response to immunomodulatory therapy, contributed to the initial diagnostic misjudgment.

Fourth, false-negative WES results. WES has inherent limitations in detecting CNVs and certain point mutations (5, 13). In this case, both the exon 8 deletion (a CNV) and the exon 22 frameshift variant were initially missed, illustrating that a negative WES result does not exclude a genetic disorder when clinical suspicion remains high.

### 4.2 Diagnostic delay: retrospective analysis and lessons learned

Retrospectively, the favorable early response to corticosteroids and IVIG, together with the initially negative whole-exome sequencing results, contributed to a diagnostic delay of more than one year. This scenario reflects a broader clinical reality: hTTP remains underrecognized in the differential diagnosis of isolated thrombocytopenia during infancy, and adverse perinatal outcomes indicated in the family history are frequently underweighted during the initial diagnostic evaluation. The present case highlights the importance of incorporating ADAMTS13 functional assays into the routine diagnostic workflow for pediatric patients with unexplained or recurrent thrombocytopenia accompanied by a suggestive familial bleeding history, regardless of initial negative genetic sequencing results.

### 4.3 Genotype–phenotype discordance

The proband carries compound heterozygous variants predicted to cause complete loss of ADAMTS13 function from both alleles, which would theoretically be expected to produce a severe phenotype—as observed in the deceased sibling. However, the proband exhibits a comparatively mild clinical course. The following explanations are proposed as hypotheses, acknowledging their speculative nature in the absence of direct experimental evidence:

The proband harbored compound heterozygous variants in the ADAMTS13 gene, which theoretically lead to complete loss of biallelic gene function and are expected to cause a severe clinical phenotype consistent with, or even more critical than, that of the deceased sibling. Nevertheless, the proband presented with a relatively mild disease course. Given that genetic verification was not performed in the deceased sibling, whether the sibling carried an identical genotype to the proband cannot be confirmed. It is speculated that the deceased sibling likely possessed a more severe genotypic profile, such as homozygous deletion, nonsense mutation, or other combinations of compound variants that result in nearly complete abrogation of ADAMTS13 activity, which genetically determined the lethal severe phenotype. Furthermore, variable phenotypic penetrance serves as a pivotal modulator of disease severity. Multiple factors, including infectious triggers, inflammatory stress, endothelial maturity, and additional genetic modifiers, collectively contribute to the phenotypic heterogeneity of hTTP. The underlying mechanisms accounting for such genotypic and phenotypic discrepancies remain to be further validated in large-scale cohort studies.

### 4.4 Key differential diagnostic criteria: ITP vs. hTTP

The gold standard for distinguishing ITP from hTTP relies on two key investigations: ADAMTS13 activity measurement (normal in ITP; severely reduced, typically <10%, in hTTP) and molecular genetic testing (no pathogenic ADAMTS13 variants in ITP; biallelic pathogenic variants in hTTP). The principal clinical implications arising from this case are as follows. First, isolated thrombocytopenia does not exclude hTTP, even in the absence of typical hemolytic features or in the presence of apparent corticosteroid and IVIG responsiveness. Second, a family history of adverse maternal outcomes—including early pregnancy loss or neonatal thrombocytopenia with hemorrhagic death—represents a critical diagnostic clue for heritable thrombocytopenic disorders. Third, WES has a high rate of missed diagnoses for structural variants and certain point mutations; in cases with clinical suspicion for hTTP, targeted ADAMTS13 gene sequencing should be performed even when WES results are negative. Fourth, bone marrow morphology is not a specific criterion for ITP and must be interpreted in the context of platelet clustering, family history, ongoing treatment, and available genetic data.

### 4.5 Limitations, follow-up, and treatment considerations

This study has several limitations. First, the follow-up duration was relatively short, and long-term monitoring is required to clarify the patient’s persistent platelet profile and the risk of acute thrombotic microangiopathy (TMA) under future stress conditions. After confirmation of the hTTP diagnosis, the patient received gradual glucocorticoid tapering, with platelet counts stably maintained at 30–105 × 10⁹/L. Recombinant ADAMTS13 replacement therapy has not been initiated thus far due to limited medical resources and drug accessibility.

Second, the therapeutic strategy warrants specific clarification. The patient was initially misdiagnosed with immune thrombocytopenia (ITP) owing to the presentation of isolated thrombocytopenia, favorable glucocorticoid responsiveness, and absence of typical hemolytic manifestations, and therefore received glucocorticoid therapy in accordance with standard ITP management protocols. After the definitive diagnosis of hTTP was established, the family was fully informed that regular prophylactic fresh frozen plasma (FFP) infusion constitutes the standard maintenance therapy for hTTP in the absence of recombinant ADAMTS13. However, the family declined long-term prophylactic FFP administration due to practical constraints, including economic burden, difficult venous access, and poor anticipated long-term adherence. Accordingly, an individualized compromise strategy was adopted: glucocorticoids were gradually tapered and discontinued under close surveillance, and on-demand FFP infusion was implemented as an alternative to regular prophylaxis. Notably, glucocorticoids are not recommended for maintenance treatment of hTTP. The current regimen represents a pragmatic choice constrained by initial diagnostic error and real-world clinical conditions, rather than an ideal standard therapeutic approach.

Third, the genotype of the deceased sibling remains undetermined. As a single-case study, the conclusions drawn herein require further validation in large-scale clinical cohorts.

## 5 Conclusion

This case shows that hTTP may manifest solely as isolated thrombocytopenia absent from typical microangiopathic hemolysis, and positive response to glucocorticoids cannot exclude this disease.For children with idiopathic thrombocytopenia and a relevant family history, even if the initial whole-exome sequencing (WES) screening is negative, it is still essential to promptly perform ADAMTS13 activity assays and targeted gene sequencing.

## Data Availability

The raw sequencing data generated for this study have been deposited in the CNGBdb Sequence Archive (CNSA) under accession number CNP0009724. The data are accessible under controlled access in compliance with the regulations on human genetic resources.
